# Addressing the overuse-underuse paradox in healthcare

**DOI:** 10.1007/s11019-025-10287-2

**Published:** 2025-07-31

**Authors:** Bjørn Hofmann

**Affiliations:** 1 Centre of Medical Ethics, University of Oslo, Oslo, Norway; 2 Institute for the Health Sciences, Norwegian University of Science and Technology (NTNU), PO Box 1, Gjøvik, N-2802 Norway

**Keywords:** Paradox, Overuse, Overactivity, Excess, Underactivity, Underuse, De-implementation

## Abstract

There is a basic contradiction in modern healthcare: while there is an urgent need for more resources to provide documented effective care in many health systems, the same systems provide extensive services that are reported to have little or no effect on people's health. This induces long wait times, delayed diagnoses and treatments, poorer prognosis, and worse outcomes. That is, a wide range of studies have demonstrated health care systems to provide large volumes of low-value services while not being able to provide much needed high-value services. This contradiction between simultaneous overuse and underuse can be analysed in a paradox framework. Moreover, identifying the drivers of overuse and underuse can help us develop strategies to curb the problem, its implications, and free resources for reducing underuse. Hence, resolving the overuse-underuse paradox is crucial for the viability of healthcare systems: for the safety, quality, effectiveness, efficiency, and sustainability of care.

## The overuse-underuse paradox

There is a basic paradox in healthcare: while there an underuse of effective health services, there is evidence of substantial overuse of health services that have little, no, or negative net value. This paradox undermines the rationale for the healthcare services, i.e., to help as many persons with their health problems as well as possible by providing safe, effective, and efficient care. Even more, it is not sustainable, threatens the persistence of many healthcare systems, and undermines the integrity of health professionals.

Already in 2010, the World Health Organization (WHO) reported that 20–40% of global health spending was estimated to be wasteful (Organization 2010) and the Organization for Economic Co-operation and Development (OECD) has stated that up to 20% of health service spending is wasteful (OECD 2017). It has been estimated that about 10% of healthcare is considered harmful, and that 30% is of low value, i.e., wasteful, duplicative or that benefits do not outweigh harms and cost (Braithwaite et al. 2020). In the USA waste has been estimated to vary from $600 billion to more than $1.9 trillion per year, corresponding to between $1800 to $5700 per person per year (Speer et al. 2020). There is strong evidence for the widespread overuse of a wide range of specific medical services in very many countries indicating that overuse and healthcare waste is common (Berwick and Hackbarth 2012; Colla et al. 2015; Brownlee et al. 2017; Lyu et al. 2017; Oakes et al. 2019; Speer et al. 2020) and a recent scoping review including 115 studies identified waste in terms of overtreatment or low-value care (42%), failures of care delivery (24%), and failures of care coordination (14%) (Olivares-Tirado and Zanga 2023). Moreover, a seminal study identified 1350 low-value services (Soril et al. 2018).

At the same time there is extensive underuse of health services (Glasziou et al. 2017). For example, there is a documented underuse of β-blockers in heart failure and chronic obstructive pulmonary disease (Lipworth et al. 2016), of behavioral treatments for headache (Langenbahn et al. 2021), and underuse of a wide range of diagnostic tests worldwide (O’Sullivan et al. 2018). Additionally, a wide range of essential health services are not provided by the healthcare systems who have documented overuse because of lack of resource allocation. Scientific advances and emergent technologies have provided a range of new, advanced, and costly diagnostics and treatments, proven to be effective, but which are too expensive to be implemented and provided. Targeted treatments in precision medicine are but one example. At the same time appropriate and effective care is not provided due to lack of access and poor organization (McGlynn et al. 2003; Runciman et al. 2012; Lipworth et al. 2016; Brownlee et al. 2017). Hence, there is a substantial underuse of healthcare services (Glasziou et al. 2017).

This amounts to a basic contradiction: while there is underuse of effective medical services (Glasziou et al. 2017), there is overuse of low-value care (Brownlee et al. 2017). More precisely, *at the same time as we are doing too little of what has great value*,* we are doing too much of what has little*,* no*,* or negative value* (Moynihan and Smith 2002; Brownlee 2007; Moynihan 2012; Glasziou et al. 2013). In this study I will investigate this contradiction in terms of a paradox framework and scrutinize the implications of the overuse-underuse paradox. Then I will scrutinize some of the mechanisms nourishing the paradox in order to point to potential solutions.

First, definitions of key terms. The overuse-underuse paradox can be defined as follows: at the same time as there are many services that are safe, efficacious, effective, efficient (cost-effective) that are not provided (underuse), there are many services that are not documented to be safe, efficacious, effective, or efficient (cost-effective) or that are documented not to be safe, efficacious, effective, or efficient (cost-effective), that are provided in the same healthcare system.

Overuse is defined as “the provision of medical services for which the potential for harm exceeds the potential for benefit”(Chassin and Galvin 1998). Overuse is often discussed and measured in terms of low-value care, which is defined accordingly as “an intervention in which evidence suggest it confers not or very little benefit for patients, or risk of harm exceeds probable benefit or, more broadly, the added costs of the intervention do not provide proportional added benefits” (Scott and Duckett 2015).

There are many types of overuse and underuse, as illustrated in Table 1.

**Table 1:** Overview of types of overuse and underuse

Overuse	Underuse
**General definition**: “the provision of medical services for which the potential for harm exceeds the potential for benefit”(Chassin and Galvin 1998). **Low-value care**: care with little or no benefit for patients or more harm than benefit	**General definition**: Failure to provide care when it could have provided a favorable outcome
**Over-investigation**: Diagnostics or referrals that are reasonably certain will not provide meaningful information (low-value examinations)	**Preventive underuse**: Failure to provide recommended preventive services, such as vaccinations, screenings, and lifestyle interventions that could prevent disease onset or progression
**Overdiagnosis**: Diagnosing a patient without symptoms with a disease that would otherwise never cause symptoms or lead to death	**Diagnostic underuse**: missed diagnosis or insufficient diagnostic testing or evaluations that are necessary to identify a condition accurately
**Overtreatment**: Treatment that does not provide benefit, or leads to more harm than good	**Therapeutic underuse**: Failure to provide appropriate therapeutic interventions or treatments that are clinically indicated
**Overdetection**: Health-related findings in a person who does not have symptoms, where the potential benefit does not exceed the risk	**Specialist Underuse**: Not referring to or not using specialists when warranted based on patients’ medical needs
**Disease mongering**: Something is made into a disease because you have diagnostic tests or treatment for the condition. (Doran and Henry 2008) For example, cosmetic medicine, and lLow testosterone (Schwartz and Woloshin 2013)	**Resource Underuse**: Healthcare resources (e.g., facilities, personnel, technology) are inadequately utilized, impacting patient access to necessary services and health outcomes
**Medicalization**: explaining and treating people’s ordinary life experiences as medical problems	

## Paradox as an analytical framework

Etymologically, the term paradox stems from Latin and Greek, meaning “contrary to expectation, unbelievable, against/beyond belief.” Paradoxes come in many kinds, e.g., as resolvable paradoxes, antinomies, and aporias (Hofmann 2001).

Resolvable paradoxes are apparent contradictions that can be resolved, e.g. by explanations or actions. Antinomies appear when (unreconcilable) principles contradict each other. Lastly, aporias are contradictions that are fundamentally unsolvable (Hofmann 2001).

It may be argued that the overuse-underuse paradox is an aporia, as no diagnostic tests are perfect, resulting in false positive and false negative test results implying inevitable overuse and underuse of health services. However, this does not hold, as accuracy (and actionability) of diagnostic tests have increased substantially, which should reduce and not increase the paradox.

Looking for potential solutions, this article will analyze the overuse-underuse paradox in terms of antinomies and resolvable paradoxes. The latter can result from linguistic vagueness, unclear concepts (e.g., what we mean by terms like “health”), incomplete or insufficient arguments, dissimilar treatment of similar cases, or the implicit balancing of interests.

## Implications of the paradox

The overuse-underuse paradox has implications for patients, professionals, the healthcare system, and society at large. These implications provide ample reasons and motivation to address the paradox.

### Patients

Overuse results in long wait times (Luigi et al. 2013; Barua 2017; Van Nynatten and Gershon 2017; Liddy et al. 2020; NuffieldTrust 2023) and reduced access to care, ensuing delayed diagnosis, prolonged anxiety and fear in patients for what causes their symptoms and suffering, as well as poorer prognosis (Ball et al. 2015; Olivares-Tirado and Zanga 2023).

Moreover, overuse results in people being diagnosed for conditions that never would have bothered them (overdiagnosis) (Welch et al. 2011). Overdiagnosis has been documented in a wide range of fields (Jenniskens et al. 2017) and subsequently results in overtreatment, as well as harms from overdiagnosis and overtreatment (Esserman et al. 2014).

Additionally, overuse may result in incidental findings of uncertain significance (Wagner and Aron 2012), sending patients on diagnostic odysseys (Hofmann and Welch 2017). At the same time, overuse may also result in worsened prognosis or shortfall, e.g., if a negative excessive test gives false assurance making patients refrain from contacting the health care system or health professionals to pursue the issue (“the recent mammogram was negative, so I should not worry about the lump in my breast”).

Underuse, on the other hand results in ignored or delayed diagnosis, poorer prognosis, increased pain, dysfunction, and suffering. Thus, underuse can as overuse result in poorer health outcome. Moreover, overuse (for some) can result in underuse (for others) in terms of opportunity costs.

Hence, both overuse and underuse can have severe implications for patients, breaching with basic principles in biomedical ethics (Beauchamp and Childress 2019), such as non-maleficence (harm and reduced patient safety, e.g. in overdiagnosis and overtreatment), beneficence (reduced prognosis, shortfall), justice (opportunity costs), and autonomy (due to lack of understanding and voluntariness).

### Professionals

Professionals may be frustrated for not providing adequate care when they cannot offer patients what is documented to be the best healthcare options due to lack of resources. This may result in a cognitive and moral burden. Additionally, they may undermine trust in the profession when not providing warranted and affordable services (underuse).

Professionals may also undermine their integrity when providing unnecessary, ineffective, inefficient, or even harmful services. However, one reason why this latter implication is not always as expressed as the implications of underuse, is because of what has been called “the popularity paradox,” (Welch et al. 2011), i.e., when people are overdiagnosed and overtreated but think that they have been helped or saved. Relatedly, when expectations (or demands) are high, people may feel that their rights are violated if they are not provided with the expected services, even if they are futile or even harmful.

Overuse may result in increased workload and poorer quality of care. Additionally, health professionals are rarely blamed or impeached for doing too much, but much more often for doing too little. That is, while underuse is overt, overuse may be covert– sustaining and enhancing the implications. Moreover, underuse may result in professional or financial penalties while overuse often gives professional and financial awards.

Thus, the overuse-underuse paradox has implications for professional integrity and trust in health professionals. The relationships are asymmetrical: overactivity may increase trust in health professionals while underactivity may reduce it, even in cases where the consequences are equally severe. Correspondingly, underactivity may undermine professional integrity while overactivity can increase it. The drivers of this are explained below.

### The healthcare system

Likewise, it is challenging for the healthcare system not being able to provide effective, safe, and efficient care. This may result in reduced trust in the healthcare system.

On the other hand, overuse is detrimental to the system as it provides poor quality services. For example, tests with low pre-test probability have low post-test validity, reducing the value of the tests. While overuse could result in reduced trust, it may not raise the same level of concern as in the case of underuse due to the described popularity paradox.

Hence, the overuse-underuse paradox has negative effects on the safety, quality, effectiveness, efficiency, and sustainability of the healthcare system.

### Society

Finally, both underuse and overuse result in poorer health in the population (than appropriate care). This may again result in reduced support for the welfare system (either it is private or public).

Overuse may have substantial opportunity costs and downstream costs, e.g., due to harms from overdiagnosis and overtreatment. Overuse also drains resources for other welfare services and for research and development of effective services. Again, decreased trust may be a result.

Conversely, underuse has negative consequences for the health of individuals and populations, undermining the welfare of societies.

Table 2 provides a summary of the implications of the overuse-underuse paradox.

**Table 2:** Implications of the overuse-underuse paradox

	Underuse (lack of provision of documented effective care)	Overuse (diagnostic, therapeutic)
Patients	Delayed diagnostics and treatment Prolonged anxiety and fear Worsened prognosis / Shortfall Infringement of rights to effective healthcare services Reduced trust in healthcare professionals and the healthcare system	Overdiagnosis and Overtreatment Harms from overdiagnosis and overtreatment Unnecessary anxiety and fear Incidental findings of uncertain significance, diagnostic odysseys Disappointment (Unrealistic expectations)
Professionals	Frustration for not being able to provide good care Responsibility: moral, financial Reduced trust in healthcare professionals	Undermined professional integrity Increased workload Waste of limited time and resources Reduced trust in healthcare professionals Cognitive and moral burden
Healthcare system	Not being able to provide relevant care Reduced trust / belief in healthcare	Providing poor quality services Waste of limited time and resources Reduced trust
Society	Poorer health Reduced support for the welfare system Decreased trust	Poorer health Reduced support for the welfare system Decreased trust

## Drivers of the overuse-underuse paradox and its mechanisms

Certainly, there are many potential explanations for the overuse-underuse paradox. For example, the scientific and technological development is much faster, more extensive, and expensive than the economic system’s resource allocations. Industry is a powerful agent in promoting overuse (Pathirana et al. 2017; Cupit et al. 2023). Overuse may be difficult to identify, measure, and target. Moreover, healthcare organizations are complex and grueling to govern. Professional autonomy may make it hard to harness overuse. Politicians may prioritize in ways that are incoherent with health professional goals and that fuel overuse. Health organizations may be incompetent that are not able to govern the services.

However, many more and complex drivers can be identified on all levels (Saini et al. 2017). To target measures to address the paradox, these drivers deserve closer scrutiny.

### Patients and the public

Individual patients are more “informed” and demanding than ever. In particular, people have great expectations to be provided with tests and treatments. This comes together with a general trend of individually oriented rights-thinking, which can drive overuse. Moreover, there is a general expectation for the healthcare system to solve a broad range of problems, not always clearly related to mental or somatic conditions. Medicine is considered a quick fix for society’s ills (Aziz 2018).

There is also an overestimation of benefits and underestimation of risks of health services, especially for advanced technology (Hofmann and Skolbekken 2017). This comes together with a general overreliance on technology (Hofmann 2015; Herstek and Shelov 2021) inciting a broad spectrum of overuse (Kriger 2014; Shanmugasundaram and Tamilarasu 2023).

On the other hand, underuse may stem from mute patient groups where vulnerable patients lose to more outspoken and strong patient groups with active (and professional) advocates. In the policy analysis literature, this is referred to as “the silent loser problem”(Weimer and Vining 2017). Moreover, certain conditions may be rare, lack knowledge, and gain little public attention. Some conditions (and specialties) also have lower prestige (Album and Westin 2008) giving patients less attention and priority than they deserve. Some patients may also experience underuse due to aesthetic stigma and discrimination, e.g., persons with obesity (Hofmann 2023a).

All the drivers identified here are resolvable paradoxes, that is they can be addressed by providing more information, adjust expectations, introduce shared decision making, reduce stigma and increase justice. Hence, the drivers direct us towards potential solutions. See below.

### Professionals

Professionals tend to be more afraid of overlooking than to overdoing in what has been called commission bias and aversion asymmetry (Hofmann 2020), according to which “the fear of omission” is worse than “the fear of commission”. This is connected to the fear of litigation and defensive medicine, being strong drivers of overuse (Hendee et al. 2010; Ooi 2020; Pausch et al. 2020; Strobel et al. 2023). The same goes for uncertainty intolerance (Hillen et al. 2017; Reis-Dennis et al. 2021), which comes together with a general thinking that it is “better to be safe than sorry” and what has been called “anticipation decision regret” (Tymstra 1989). This is a resolvable paradox, as biases and uncertainty intolerance are manageable issues.

Additionally, there is a professional strategy to “make something happening” (*ut aliquid fiat*) in order to satisfy patient (and proxy) expectations. Withholding or withdrawing services is perceived of as undermining the professional identity (Hofmann and Lea 2023). Moreover, while clinical guidelines may ascertain appropriate care, they may also drive overuse, especially when developed by sub-specialist influenced by spectrum bias (Litkowski et al. 2016).

Furthermore, lack of skills and competency can spur both underuse and overuse. In the former case it may be due to a lack of skills in diagnostics and treatment. This is especially prominent for rare diseases or untypical diseases. In latter case, lack of skills in clinical examination can incite the reliance on and use of diagnostic tests. However, as alluded to, diagnostic tests without thorough clinical examination, lowers the pretest-probability, and thereby the validity of a test result (Doubilet 1988), which in turn drives additional tests. Instead of decreasing uncertainty it increases uncertainty, and thereby spurs a series of additional tests. Moreover, instead of developing clinical skills it upsurges reliance on diagnostic tests establishing a self-reinforcing circle (Kraemer 1992; Maynard and Frankel 2006). Again, this is a resolvable paradox as skill training may be address the issue.

Additionally, health professionals tend to share the asymmetry of risks and benefits with the general population (Hofmann 2019a, b). They tend to overestimate the benefits and underestimate the risks of healthcare interventions, especially when they involve advanced technology (Hofmann 2019a, b). The professional identity of health professionals is also formed by activity (and not by inactivity)– by providing services, not by withholding them (Cruess and Cruess 2016). See above on *ut aliquid fiat*.

Correspondingly, overuse may be spurred by the need to obtain and maintain professional skills. Low volume is related to low quality (Halm et al. 2002) and for specific professional specialties certain number of procedures are required. Hence, the need for training may spur medical overactivity.

Additionally, certain conditions, procedures or services have low professional status or prestige and therefore gain less attention (Album and Westin 2008; Album et al. 2017). Thus, professionals drive both underuse and overuse through a range of mechanisms. Here I have only mentioned a few relevant for the task of addressing the paradox. However, all drivers are addressable rendering it resolvable paradox.

### Industry and technology

Hyping scientific progress and the quest for profit are obvious drivers for medical overuse (Moynihan et al. 2002, 2013; DeAngelis and Fontanarosa 2008). However, more subtle processes are also at play. For example, diagnostic industry is providing technology with increased accuracy (e.g., in terms of improved sensitivity and specificity) and actionability. This results in detecting more and milder cases, which provide better outcomes when treated. This is considered as a success, spurring further improvements in accuracy. The process is enhanced by lowering thresholds and cutoffs, increasing the prevalence of a wide range of conditions and the treatment of ever milder cases. Increased prevalence, upsurges attention and allocated resources. However, the result may only be extended overdiagnosis and overtreatment (Welch et al. 2011; Hofmann and Welch 2017).

Technological advances are also used to define (Moynihan et al. 2002) and expand disease definitions (Hofmann 2018, 2019a, b). Diseases are ever more defined in terms of biomarkers and less based on symptoms and first-person experiences, such as pain, reduced function, and suffering. Alzheimer’s disease is but one example of a condition being defined by biomarkers and not symptoms or experienced illness (Alzheimer’s Association Workgroup 2023; Karceski and Antonopoulos 2023) and where industry is strongly involved in defining the disease (Widera 2024). A weak correlation between the biomarker and suffering is a strong driver of overuse.

Industry has also been documented to apply a range of measures to widen the concepts of disease and aggressively expand the markets for their diagnostic or therapeutic products in what has been called disease mongering (Payer 1992; Moynihan et al. 2002; Dear and Webb 2007; Doran and Henry 2008). Additionally, industry every year uses substantial resources to influence clinicians, health care managers, and health policy makers to promote the use of their products (Burns 2012; Meyers et al. 2021; Schnog et al. 2024). Moreover, industry tend to follow the money (and the volume), biasing the innovation towards large patient groups and big revenues– contributing to underuse for small and non-profitable patient groups.

In sum, technology development and industry are forceful drivers for the overuse-underuse paradox. While some aspects are resolvable, such as unwarranted expansion of diagnostic testing and disease definitions, others are antinomies, as they stem from genuine conflicting interest.

### Healthcare system

Healthcare systems are characterized by silo organization, where lack of communication is a clear driver for the paradox. For example, when a radiologist wants to discuss an inappropriate referral, there is no smooth or effective way to communicate with the referrer. It is much easier to perform the examination than to stop it or to ask for more information (Brandsæter et al. 2023).

Correspondingly, the financing (reimbursement) system also spurs overuse. Activity is rewarded (or reimbursed), while inactivity is not as there are traditionally no task or reimbursement codes for halting unnecessary examinations or treatments. On the contrary, there are active systems for sanctions in the case of underuse. These economic incentives come together with increasing time pressure and demands for efficiency.

Yet another driver is access to services. According to Roemer’s law “A built bed is a filled bed,” (Roemer 1961), i.e., the health care services are strongly supply-sensitive (Fisher and Wennberg 2003; Wennberg 2010). Even more, the fact that the alternative costs and the alternative opportunities are mainly covert to healthcare providers makes it difficult to harness overuse. Underuse, on the other hand, are frequently highlighted, e.g., by the media.

The strongest drivers of underuse are clearly lack of knowledge (of specific diseases), skills (to diagnose and treat them), and shortage of allocated resources. However, as pointed out, mute and vulnerable groups may be underserved. Relatedly, *the inverse care law* can explain part of the paradox: “The availability of good medical care tends to vary inversely with the need for it in the population served. This inverse care law operates more completely where medical care is most exposed to market forces, and less so where such exposure is reduced. The market distribution of medical care is a primitive and historically outdated social form, and any return to it would further exaggerate the maldistribution of medical resources.” (Tudor Hart 1971).

While some aspects are resolvable, e.g., by reorganizing the healthcare system, others are based in conflicts of interests, e.g., increased population health versus cost-effectiveness versus obtaining political goals etc.

### Society/Culture

On a cultural level, there are several trends impelling medical overuse. Health has come to be considered a value in itself and a goal in life (Clerc 2004; Saad and Prochaska 2020), making people seek all measures to promote and enhance health. This comes together with a strong belief in the omniscience and omnipotence of medicine (Porter 1999). Accordingly, the healthcare system is considered to be an efficient problem solver and is therefore approached to solve a wide range of social problems (Aziz 2018) (e.g., children’s ability to adapt an unstimulating school environment) (Mather 2012).

The strong (but controversial) (McKeown 2005) belief in medicine is enhanced by a general belief in science and technology (Hofmann 2002, 2019a, b), which are considered to be general problem solvers. This is spurred by biases such as “advanced is better than simple,” “more examinations are better than few,” “early testing is better than late,” and “better to be safe than sorry” (Hofmann 2019a, b).

On the other hand, there are also some cultural drivers of underuse. For example, distrust and skepticism to specific health services may result in underuse. Vaccine skepticism (Tafere et al. 2024) is but one example. Austerity may be another reason.

While several of the social and cultural drivers may be resolvable, some of them origin in value conflicts and in polarized debates (Hofmann 2024a, b). Hence, they are antinomies. Additionally, some may also be considered to be irresolvable, i.e., aporias.

Table 3 provides a summary of some of the many drivers of the overuse-underuse paradox. Clearly, this overview is by no means exhaustive and exclusive. The point here is to motivate and elaborate the measures to resolve the overuse-underuse paradox.

**Table 3:** Drivers of the overuse-underuse paradox and measures to resolve it

Drivers of the overuse-underuse paradox	Measures to resolve the paradox
Patients and the public: - Demand - Expectation that the healthcare system will solve problems - Overestimation of benefits and underestimation of risks - Overreliance on technology	Patients and the public: - Provide reality checks, education - Align expectations to documented benefits and risks - Help people to differentiate between hype and reality
Professionals: - Aversion asymmetry - Uncertainty intolerance - Strategies to make something happen (“ut aliquid fiat”) - Lack of skills and competency - Asymmetry of risks and benefits	Professionals: - Promote professional integrity - Facilitate uncertainty management - Communicate the value of non-action - Improve clinical skills, gatekeeping - Avoid ambiguity/uncertainty aversion and other biases
Industry and technology: - Increasing accuracy (sensitivity and specificity) and actionability - Lowering thresholds and cutoffs - Defining and expanding disease	Industry and technology: - Outcome-based assessment of technology - Harness unwarranted thresholds - Limit inappropriate expansion of disease
Healthcare system: - Silo organization - Financing (reimbursement) system - Efficiency and time pressure - Access (Roemer’s law) - Ignorance of alternative costs	Healthcare system: - Improve stakeholder communication - Adjust financing systems (caps) - Regulate access, Proper evaluation - Visualize alternative costs - De-implementation, disinvestment
Society/Culture: - Health as a value and goal in life - The healthcare system considered to be a problem solver - General belief in science and technology	Society/Culture: - Relativize the value of health - Limit healthcare measures to its subject matter (where efficient) - Reality check the belief in science and technology

## Measures to resolve the overuse-underuse paradox

Many measures have been introduced to reduce medical overuse (Polisena et al. 2013; Kamaruzaman et al. 2022; Cupit et al. 2023). De-implementation (Walsh-Bailey et al. 2021), reassessment (MacKean et al. 2013; Soril et al. 2018), disinvestment (Orso et al. 2017), as well as a range of professional initiatives, such as Choosing Wisely (Schpero 2014; Levinson et al. 2015), and Preventing Overdiagnosis (Moynihan et al. 2012) are but a few examples. However, reducing overuse and underuse has turned out to be challenging (Haas et al. 2012; Harris et al. 2017b; Mitchell et al. 2019; Rotteveel et al. 2021; Pu et al. 2023). One reason for this is that many drivers are covert, inert, complex, and related to basic human biases (Hofmann 2020). Nonetheless, there are ways to mitigate overuse (Pathirana et al. 2017; Cupit et al. 2023) and to address the overuse-underuse paradox.

As pointed out above, many of the drivers of the overuse-underuse paradox are resolvable or antinomies. Hence, they can be handled. An overview of measures to reduce overuse and underuse is provided on the right column of Table 2.

### Patients and the public

Providing reality checks for people’s expectations and making visible the consequences of unwarranted demands may be good strategies for reducing overuse and free resources for targeting underuse. For example, showing people how the overuse of other health services may be as harmful as the overuse of antibiotics, may increase awareness. The general attitude to take an extra X-ray just to be safe may make you sorry when the resulting wait time delays diagnosis and worsens prognosis. Information campaigns towards the public (and professionals) like the Choosing Wisely Initiative is an important example of such measures (Blumenthal-Barby 2013; Schpero 2014; Soril et al. 2018; Cliff et al. 2021). However, understanding the described drivers can also help addressing underuse, e.g., by providing more information about available effective care.

Another strategy to reduce overuse is to align expectations to documented benefits and risks of health services and to help people to differentiate between hype and reality. More trustworthy reports of outcomes and well-organized shared decision-making are good examples (Han et al. 2021). The strong drivers of overuse (including industry using extensive resources for promotion) indicate that the task is not easy. However, to identify individual cases of overuse (e.g., overdiagnosis and overtreatment) and reveal the negative consequences to the general public, may be one way to increase awareness. Informing the Norwegian public about excessive radiological examinations is one example (Hofmann 2021).

On the other hand, to inform about the rights to care is a way to mitigate underuse of health services. However, we should be careful to expect patients to know which services are effective and efficient and to which they are entitled.

### Professionals

Improving competency and clinical skills (Hasanpoor et al. 2020; Kool et al. 2023), promoting professional integrity, and facilitating uncertainty tolerance (Han 2021) are but a few relevant strategies to resolve the paradox. The same goes for communicating the value of non-action in comparison to action. Even more, facilitating the gatekeeping functions of professionals (giving them credit instead of punishment) are important measures to avoid professionals driving the overuse-underuse paradox.

Professional initiatives, such as Choosing Wisely, have identified overuse in a wide range of specialties and services (Soril et al. 2018) and suggested specific and targeted measures to reduce it. However, documented outcomes from such initiatives are moderate (Cliff et al. 2021). One reason for this is that measures have not been able to take the complexity of the healthcare system into account. Systematic reviews show that combined interventions that facilitate interprofessional communication are the most efficient to reduce overuse (Colla et al. 2017; Kjelle et al. 2021).

Additionally, avoiding tendencies amongst professionals, such as ambiguity aversion, uncertainty intolerance and other biases are crucial measures to reduce or resolve the overuse-underuse paradox (Hofmann 2019a, b). While by no means easy, educational and practical skill training measures as well as decision-support systems are important measures.

Focus on professional integrity and societal trust is also important to avoid both under- and overuse. This is a task for professionals and professional organizations.

### Industry and technology

Harnessing industry is clearly not an easy task. However, the mission is more to direct their activity away from overuse (and underuse) and towards high-value care. Hence, directing revenues away from low-value towards high-value care is an important strategy, i.e., reducing profit from overuse and increasing profit from targeting underuse. This can be done by stimulating outcome-based assessment of and payment for technology and services as well as more frequent and strict reassessment, outcome monitoring, and active disinvestment (MacKean et al. 2013; Orso et al. 2017).

As industry has defined and stimulated the use of ever more indicators (biomarkers, predictors, precursors)(Hofmann 2018) that are more remotely connected to experienced pain, dysfunction, and suffering, it is crucial to move the assessment of outcomes from surrogacy measures to “hard outcomes” (PROMS and PREMS) (Hofmann 2018, 2019a, b). It is imperative that the health services are directed at what matters to people’s experienced health (Hofmann 2024a, b). Accordingly, harnessing threshold and cutoff creep, as well as limiting inappropriate expansion of disease (Hofmann 2018, 2019a, b) and indication creep (Djulbegovic and Paul 2011) are other essential strategies that target key drivers (see above).

While the unwarranted ties between clinicians and industry hve been reduced, the influence from industry deserves continuous monitoring.

### Healthcare system

Correspondingly, to improve communication between actors and stakeholders is an important strategy to hamper the silo-effects fueling the overuse-underuse paradox. The same goes for adjusting the financing systems, e.g. by directing reimbursement towards high-value outcomes (Miller 2009). Setting caps on low-value services is but one measure. Again, a range of reassessment (MacKean et al. 2013), disinvestment (Harris et al. 2017; Harris et al. 2017a, b, c) and de-implementation frameworks (Morgan et al. 2017; Walsh-Bailey et al. 2021) are available.

A highly potent measure to reduce overuse and direct resources towards underuse is to regulate resources and restrict access to services. While this directly addresses Roemer’s law, it is controversial and politically provocative. A less contentious approach is to elaborate and visualize the consequences of the paradox and its alternative costs. Envisioning who will not get care as a result of overuse is potentially effective.

Another measure to resolve the overuse-underuse paradox is to limit healthcare measures to its subject matter, such as individuals’ pain and suffering (Hofmann 2023c; Hofmann 2024a, b). Avoiding the expansion of medicine and its responsibility to areas beyond human bodily and mental issues may reduce the paradox. For example, containing medicalization, overdiagnosis, and overtreatment may make significant contributions (Bell and Figert 2012).

### Society/Culture

On the societal level it may be fruitful to avoid health anxiety, healthism, and to stop the expansion or enhancement of health (Hofmann 2023). As more attention and resources has been directed towards persons without symptoms, less means become available for the underserved. “An ounce of prevention is worth a pound of cure” only when the prevention is effective.

Yet another measure to resolve the overuse-underuse paradox is to foster uncertainty tolerance in the general population (Dugas et al. 1997; Hillen et al. 2017; Strout et al. 2018; Han 2021) (as amongst professionals). Actively preventing the worried well (Wagner and Curran 1984) to capture the attention and resources from the underserved silent sufferers is a crucial strategy that requires public awareness and solidarity.

Additionally, facilitating reality checks for the belief in science and technology may be important measures to reduce the overuse-underuse paradox and to improve the sustainability of the health services. Informing the general public about the unintended harms from good intentions (Slawson and Shaughnessy 2021) is a crucial measure.

Being open about limited resources and the necessity for priority setting can also reduce the paradox but this may be difficult on a political level.

## A short case study: radiology

The overuse-underuse paradox comes out clear in radiology where there has been a substantial overuse of imaging services at the same time as there are long wait lists for examinations. Overuse has been documented in terms of low-value imaging, over-investigation, overdetection, and overdiagnosis (with subsequent overtreatment) (Hofmann 2025). Additionally, there has been therapeutic use of diagnostic imaging (anxiolytic use) (Park et al. 2021). More than 100 examinations have been identified as overuse (Levin and Rao 2017) ranging from 20 to 100% low-value imaging (Kjelle et al. 2022) globally amounting to a cost of between 66 and 166 bn USD (Kjelle et al. 2024).

At the same time there is an underuse of radiological services (Soneji et al. 2017; England et al. 2021). There is preventive underuse in several screening programs, e.g., in mammography screening where high-risk groups are underrepresented (Schwartz et al. 2021). Moreover, there is diagnostic underuse as there is a lack of access to services in many countries, and delayed access due to overuse (Nuti and Vainieri 2012; Van Nynatten and Gershon 2017). This has severe consequences in terms of delayed diagnosis and treatment and poorer prognosis.

Drivers of the overuse-underuse paradox in radiology has been documented amongst stakeholders (radiologists, referrers, and patients) and are related to organization, communication, competence, expectations, defensive medicine, roles and responsibilities, and referral quality and time constraints (Brandsæter et al. 2023). One common mechanism is that the referrer refers a patient to radiology despite the lack of medical indication to maintain a good relationship, hoping that the radiologist will stop imaging due to lack of appropriateness. The radiologist acknowledges the inappropriateness but does not know the patient and does not have enough information. Moreover, stopping the examination results in increased responsibility and loss of income. Therefore it is much easier to perform the examination (Brandsæter et al. 2023).

While most of the drivers point to issues that are resolvable, some are antinomies as they stem from conflict of values or interests, e.g., the interest of maintaining good relationship with patients and income or activity versus quality and safety. Biases, such as a greater fear of overlooking than overdoing (aversion asymmetry) (Hofmann 2019a, b) also point to conflicts between System 1 and System 2.

A wide range of interventions have been suggested to mitigate and resolve the overuse-underuse paradox in radiology. Increased access and more appropriate imaging are measures to reduce underuse. Decision-support systems, clinical guidelines, education, audits, and feedback are amongst the most applied interventions to reduce overuse (Kjelle et al. 2021). One reason why the success of many interventions to reduce overuse (and underuse) is moderate is that underlying conflicts of interest have not been properly acknowledged and addressed, i.e., that antinomies are not recognized and handled.

## Discussion

This article has defined and investigated what has been called the overuse-underuse paradox. Moreover, it has identified a series of its drivers in order to point to potential strategies to resolve the paradox. The list of drivers is by no means exhaustive. Neither is the suggested strategies. Much more is provided in the referred literature. However, the point here has not been to provide an exhaustive and exclusive literature review. More details can be found elsewhere (Morgan et al. 2017, 2018; Soril et al. 2018; Cupit et al. 2023; Kool et al. 2023). The special take in this article has been to present the problem in the framework of a paradox. Showing that the paradox is neither rational nor sustainable is a way to emphasize its untenability and to direct our attention towards targeting specific and strong drivers. Moreover, the analysis of what type of paradox the various drivers represent has pointed to potential solutions.

The reassessment, disinvestment, and de-implementation literature is rich on examples of how difficult it is to reduce overuse. Correspondingly, the media is proficient in revealing underuse. Hence, although reviewing manners to mitigate under- and overuse, I do by no means suggest that there are any simple solutions. The variety and complexity of the system and the drivers indicates that there is no quick fix to resolve the overuse-underuse paradox. By addressing the implications, I have tried to provide motives to address the paradox and by focusing on the drivers, I have indicated where we can target the problem.

In this article, the problems of overuse and underuse has been analyzed in terms of paradoxes. This may represent an analytic limitation, as other analytical approaches could have been applied. For example, the issue of overuse and underuse could have been studied in terms of imbalance. This may have revealed other important aspects and potential solutions. However, it may also have led to problems in defining “balance” and reflections on whether balance is attainable. Nonetheless, the paradox framework has its limitations, as it may exclude other relevant aspects. However, it provides one way to explain the causes of the overuse-underuse problem and directing attention towards action.

Moreover, it may be argued that there is an imbalance in the referenced literature on underuse and overuse. This is because there are five times as many articles in PubMed with the Key term “overuse” than “underuse.” Moreover, the analysis has revealed that there is not one “overuse-underuse paradox” but many (that need special attention and specific solutions). However, for linguistic simplicity, I have written “overuse-underuse paradox” (in singular and not plural).

As several drivers can be classified as antinomies highlighting, aligning, or directing the interests of stakeholders can be viable strategies to resolve the paradox. In particular, bringing medicine back to its basic values and its subject matter, i.e., to reduce the suffering of individuals (Hofmann 2024a, b), is one way of aligning the interests. As acknowledged, the suggested measures are by no means easy solutions. The psychology, behavioral economics, and disinvestment literature is affluent in explaining the difficulties with changing attitudes, behavior, and organizational structures. Nonetheless, it is crucial that we focus on the measures targeting root causes to improve the healthcare system.

Clearly, the overuse-underuse paradox plays out differently in distinct healthcare systems. The drivers and strategies may be different in pay-for-performance systems than in welfare-based systems. It may also vary with the capacity of the system and access to care. Nonetheless, the overuse-underuse paradox can be observed in most the healthcare systems of most affluent countries. Moreover, this indicates that we can learn from countries where the overuse-underuse paradox is not present (so-called reverse innovation).

Admittedly, this study has itself an inbuilt bias, as overuse is connected to underuse, and overuse appears to be given more attention. However, as underscored, there are strong drivers of underuse as well: lack of resources, knowledge, skills, and access to care. Moreover, history has shown that bluntly allocating more resources has not been overly successful. It will increase overuse more than reducing underuse. Hence, we need to target overuse to allocate resources for reducing underuse.

The distinctions and categories applied are certainly neither exhaustive nor exclusive. For example, there are overlaps between aspects sorted under “patients and the public” and “society/ culture”. For instance, “expectations that healthcare system will solve problems” are related to “the healthcare system considered to be a problem solver.”

Although I have pointed to a series of strategies to address and resolve the paradox, I have said little about who is responsible for the various tasks. Clearly, the various stakeholders are responsible for tasks in their own field. However, adjusting expectations to the healthcare system could be a joint task of the population, professionals, health policy makers, and public organization. As such responsibilities may be complex and vary between healthcare systems, this issue could not be included in this study.

Definitions of key concepts, such as underuse, and overuse are widely discussed in the literature (Brownlee et al. 2017; Morgan et al. 2017) and deserve more attention than is given in this article. So does how these phenomena can be measured. In this article, I have only taken the empirical research in the field (and their pragmatic definitions) as a point of departure.

In addition to the above-mentioned limitations, the article also falls between several disciplinary stools. While it addresses the implications of the paradox, it does not investigate the ethical issues in any detail. Although the article uses paradoxes as its analytical framework, it provides no in-depth paradox analysis. While the article benefits from health services research, health policy, and implementation sciences it provides no basic examination in any of these fields. Nonetheless, the article has addressed four crucial questions: (1) what is the overuse-underuse paradox? (2) What are its implications for patients, professionals, the healthcare system, and society? (3) What are the mechanisms nourishing the paradox? (4) Given 1–3, what are potential (re)solutions of the overuse-underuse paradox?

## Conclusion

This article has scrutinized a basic contradiction in modern healthcare, i.e., the simultaneous overuse and underuse of health services, in terms of a paradox framework. While there is an urgent need for more resources for documented effective care in many health systems, the same systems provide extensive services that are documented to have little or no effect on people’s health. This has detrimental effects on patients, such as long wait times, delayed diagnoses and treatments, poorer prognosis, and worse outcomes. It also has bad implications for professionals and the healthcare system, such as reduced safety, effectiveness, efficiency, and sustainability of care. Additionally, it has social implications in terms of reduced trust in healthcare and a welfare system. In order to elaborate strategies for reducing overuse, this article has identified a range of specific drivers of overuse and underuse that interact in a complex system. While resolving the paradox is not easy, the paradox framework differentiates between resolvable paradoxes, antinomies, and aporias. Moreover, the analysis highlights the irrationality and lack of sustainability of the overuse-underuse paradox and points to common goals and strategies to reduce overuse and free resources for reducing underuse. In particular, it directs our attention to resolvable paradoxes and antinomies. Addressing, the overuse-underuse paradox is crucial for the viability of healthcare systems: for the safety, quality, effectiveness, efficiency, and sustainability of care.
